# Fatty Acids, Antioxidants and Physical Activity in Brain Aging

**DOI:** 10.3390/nu9111263

**Published:** 2017-11-20

**Authors:** Hércules Rezende Freitas, Gustavo da Costa Ferreira, Isis Hara Trevenzoli, Karen de Jesus Oliveira, Ricardo Augusto de Melo Reis

**Affiliations:** 1 Laboratory of Neurochemistry, Institute of Biophysics Carlos Chagas Filho, Universidade Federal do Rio de Janeiro, Rio de Janeiro 21941-901, Brazil; gustavolhe@gmail.com (G.d.C.F.); ramreis@biof.ufrj.br (R.A.d.M.R.); 2 Laboratory of Neuroenergetics and Inborn Errors of Metabolism, Institute of Medical Biochemistry Leopoldo de Meis, Universidade Federal do Rio de Janeiro, Rio de Janeiro 21941-901, Brazil; 3 Laboratory of Molecular Endocrinology, Institute of Biophysics Carlos Chagas Filho, Universidade Federal do Rio de Janeiro, Rio de Janeiro 21941-901, Brazil; haraisis@biof.ufrj.br; 4 Laboratory of Endocrine Physiology and Metabology, Biomedical Institute, Universidade Federal Fluminense, Niterói 24210-130, Brazil; karenoliveira@id.uff.br

**Keywords:** essential fatty acids, ascorbic acid, glutathione, aging, senescence, nervous system, growth factors, neuroprotection, docosahexaenoic acid, inflammation

## Abstract

Polyunsaturated fatty acids and antioxidants are important mediators in the central nervous system. Lipid derivatives may control the production of proinflammatory agents and regulate NF-κB activity, microglial activation, and fatty acid oxidation; on the other hand, antioxidants, such as glutathione and ascorbate, have been shown to signal through transmitter receptors and protect against acute and chronic oxidative stress, modulating the activity of different signaling pathways. Several authors have investigated the role of these nutrients in the brains of the young and the aged in degenerative diseases such as Alzheimer’s and Parkinson’s, and during brain aging due to adiposity- and physical inactivity-mediated metabolic disturbances, chronic inflammation, and oxidative stress. Through a literature review, we aimed to highlight recent data on the role of adiposity, fatty acids, antioxidants, and physical inactivity in the pathophysiology of the brain and in the molecular mechanisms of senescence. Data indicate the complexity and necessity of endogenous/dietary antioxidants for the maintenance of redox status and the control of neuroglial signaling under stress. Recent studies also indicate that omega-3 and -6 fatty acids act in a competitive manner to generate mediators for energy metabolism, influencing feeding behavior, neural plasticity, and memory during aging. Finding pharmacological or dietary resources that mitigate or prevent neurodegenerative affections continues to be a great challenge and requires additional effort from researchers, clinicians, and nutritionists in the field.

## 1. Introduction

Throughout the 20th century, evidence-based medical knowledge has allowed for a significant increase in life expectancy, especially in well-developed countries. Epidemiological data from 1900 (United States and United Kingdom) indicate that 50% of the population lived approximately until 50 years old, while in the 1990’s, half of the population lived until 80 years old [1]. The world health organization (WHO) estimates that there was a global increase of 5.0 years in life expectancy between 2000 and 2015, with an even larger increase of 9.4 years observed in Africa (WHO, 2016). The aging process is, however, permissive for the development of several degenerative disorders and infectious diseases, which are strongly influenced by nutritional imbalances, inflammation, metabolic exhaustion, and the natural process of cellular senescence [2].

Insufficient ingestion and/or deficient absorption of essential nutrients deeply affects the health conditions of elderly individuals. Frangeskou and coworkers explored the impact of dehydration as an extenuating factor for public expenses with health services, increasing mortality, hospital readmission, and period of stay under medical/hospital care [3]. Digestion and absorption of nutrients is normally deficient in the elderly, as compared with younger individuals. In a recent study, it was shown that essential and branched-chain amino acids reach peak blood levels within 1 h after young individuals (20–25 years old) receive a protein-rich meal, while the same peak concentrations were reached only 3 h post-meal for an elderly (60–75 years old) group [4].

The prevalence of malnutrition, weakness, and related disabilities are also relevant factors and may encompass a large portion of the aged population, mainly those institutionalized (hospitalized) and resident of non-developed/developing countries [5]. In a cross-sectional Brazilian epidemiological study with elderly individuals (≥60 years old), anemia index, hemoglobin concentration, and population frailty were intrinsically related, indicating that low levels of hemoglobin are associated with a greater number of frailty indicators (Fried phenotype criteria) [6].

On the other hand, the consumption of Western diets is increasing worldwide, and these diets are characterized by high lipid consumption (mainly saturated fatty acids, or SFA) and refined carbohydrate consumption with low ingestion of vegetables, and they have been associated with the development of obesity, cardiovascular disorders, cancer, and diabetes [7]. Loss of endothelial homeostasis during aging, for example, strongly depends on nutritional factors, oxidative stress, and inflammation. Dietetic interventions in elderly people are, however, hardened by cognitive impairment and loss of mobility, which limits their autonomy for preparing complex meals, as well as for chewing and digesting food [8]. Regulation of the circadian cycle and a decrease in dietetic calories content has been shown to effectively favor longevity in several in vivo models [9].

In emerging countries, such as Asian and Latin-American nations, it is possible to observe a marked effect of nutritional transition, parallel to the accelerated expansion of urban areas, which incorporates deleterious dietary habits in the population [10]. This factor introduces a deep epidemiological concern, since modifications in feeding habits and obesity are strong indicators of health risk, such as high blood cholesterol, pre-diabetes, hypertension, asthma, arthritis, and bad or regular self-reported health condition [11]. Weight variations affect the well-being of elderly patients, a determinant factor for survival within this group [12].

Depression may also be related to the development of obesity [13], and obesity itself is significantly associated with alcohol abuse and depression, mainly in adult female individuals or highly obese subjects [14,15]. Current dietetic approaches rely on providing balanced amounts of energy, and macro and micronutrients; other therapies, such as correction of the gut microbiome and global intestine health, await further clinical evidence [16]. Here, we explore post-transitional aspects of modern feeding, especially the intake of fatty acids and antioxidants, which greatly relate to the process of brain aging, one of the pillars in generalized senescence.

## 2. Senescence of the Nervous System

Many disabling central nervous system (CNS) symptoms and diseases are highly associated with the aging process, including cerebrovascular disease, Alzheimer’s disease (AD), and Parkinson’s disease (PD), as well as decline in attention and memory [17,18]. Despite the current medical advances to extend lifespan, untangling the precise metabolic interactions involved in the process of neural aging continues to be a challenge. Both endogenous and environmental factors have been postulated to trigger cellular senescence, including genetic alterations (DNA damage and shortening of telomeres) and non-physiological activity of transcription factors [19,20], accumulation of aberrant proteins [21], excitotoxicity [22], oxidative damage, mitochondrial dysfunction [23,24], and others. Here we focus mainly on stress-induced senescence, aiming to pinpoint the aspects of aging in the context of food behavior and physical activity in modern (urban) societies.

It has been shown that the disturbances in brain synaptic circuitry that occur, especially in the hippocampus and pre-frontal cortex during aging, might promote relevant cognitive decline [25]. Oxidative damage accumulates with age and is potentially harmful to many mitochondrial functions. Contributing factors include decreased membrane fluidity and the intrinsic rate of proton leakage across the inner mitochondrial membrane [26]. Previous reports showed that mitochondria are chronically depolarized in senescent neural cells; this includes an age-dependent decrease in mitochondrial membrane potential in cerebellar neurons from brain slices [27] and in cultured basal forebrain neurons [28]. Brain mitochondria from senescent rats present damaged mitochondrial complex I, which may be related to the increase of Bax/Bcl-2 cascade (apoptosis regulator) observed in these mitochondria [29]. It has also been shown in rats and humans that mitochondria from senescent subjects are larger than in younger subjects, but fewer in number [30,31,32]. On the one hand, the total volume of the cell occupied by mitochondria is virtually unaltered in young and older subjects, and these larger mitochondria do not feature the same bioenergetic capability [32,33]. Potential consequences of mitochondrial chronic depolarization include impaired ATP synthesis and redox homeostasis, as well as disruption of the calcium gradient across the mitochondrial membrane with subsequent impairment of calcium stores or increase in the threshold necessary to trigger cation uptake [34]. Thus, changes in the metabolic status would greatly impair the fuel reserves of the neural cells and consequently make them less capable to respond to injury. In the context of the cognitive impairment of aged subjects, the linchpin seems to be the activity of the fast-spiking interneurons [35], which have high metabolic demands and thus are more susceptible to metabolic dysfunction [36].

Accumulated data indicate that different mechanisms, including gradual dysfunction of respiratory chain complexes involved in the electron transfer (mainly complexes I and IV), flaws in compensatory mechanisms, inaccurate gene expression, and increased number of mitochondrial DNA (mtDNA) damage, may influence the progression of AD [37]. Blood glucose homeostasis and several associated metabolic pathways appear to be altered in the brain of AD patients; however, these manifestations may be consequence of the progression of aging and disease, which undermine synapses and attenuate the demand for glucose, further contributing to the functional and progressive decline of cerebral functions [38].

One of the main regulators of growth and survival in adverse environmental conditions, the mammalian target of rapamycin (mTOR), is a catalytic subunit of two distinct complexes known as mTOR1 and mTOR2 complexes (mTORC1 and mTORC2, respectively) [39]. The intrinsic communication of mTOR complexes (mainly mTORC1) with the metabolic control of glycogenesis and lipogenesis is essential to maintain central homeostasis [39,40], since neural cells are highly dependent on the continued supply of glucose and other energy substrates (e.g., ketone bodies) to maintain the ATP/AMP ratio. This dynamic allows for the correct regulation of autophagy systems, essential for the clearance of malfunctioning organelles and misfolded proteins, which have been found to be dysregulated in central diseases such as AD [41].

Nutritional profile of older individuals seems to be important to the progression of several pathological conditions affecting CNS. It has been reported that the occurrence of disabilities and signals of fatigue are significantly correlated to diet deficiency of folate (i.e., vitamin B9) and magnesium in patients with multiple sclerosis (MS) [42]. The onset of preclinical indicators for AD suggest that the availability of micronutrients and fatty acids, especially docosahexaenoic acid (DHA), is gradually restricted and follows the progression of the disease in aged subjects. Protein-energy nutritional status is also aggravated in AD, but it usually parallels the symptoms of cognitive impairment. Nutritional strategies that combine key nutrients for the formation and maintenance of synaptic integrity have been used primarily to prevent loss or impairment of memory in AD patients [43]. In vivo restriction in the supply of nutrients during pre- and post-natal periods causes metabolic changes to the blood-brain barrier (BBB), inducing cognitive disorders and predisposition to AD [44]. These findings underscore an intrinsic relationship between adequate supply of essential nutrients, especially fatty acids and antioxidants, and maintenance of central homeostasis during aging. 

## 3. Adiposity, Neuroinflammation and Senescence

The hypothalamus is a key region in the CNS controlling energy homeostasis, and it is sensitive to metabolic signals, such as fatty acids and glucose, and several hormonal signals derived from peripheral tissues such as gut, pancreas, and adipose tissue. Increased adiposity or obesity are frequently features in senescent subjects. Both aging and obesity can affect the central regulation of systemic homeostasis, increasing the risk of developing AD, insulin resistance, diabetes mellitus, and cardiovascular and cerebrovascular diseases. However, because these two metabolic conditions commonly coexist, it is difficult to distinguish the relative contribution of each one to the disease progression. Neuroinflammation seems to be a common mechanism by which these conditions independently and interactively impair neurogenesis, neural stem cell survival and differentiation, and promote aging-related cognitive decline and neurodegenerative diseases [45,46].

Neuroinflammation is associated with BBB breakdown and neurodegeneration. Because obesity is related to a persistent pro-inflammatory state [47], plasma-derived deleterious factors such as lipopolysaccharides (LPS) and SFA can pass through the damaged BBB to induce neuroinflammation. In fact, serum derived from aged mice or aged high-fat fed mice produces significant microglia activation, with increased reactive oxygen species (ROS) production and cytokine expression in the hippocampus [48]. On the other hand, Nlrp3 inflammasome knockout mice show decreased metabolic and inflammatory markers in peripheral and central tissues, improved functional cognitive decline during aging, and extended lifespan [49]. In the hypothalamus, both aging and over nutrition increase the proinflammatory axis comprising IκB kinase-β (IKKβ) and its downstream nuclear transcription factor NF-κB (IKKβ/NF-κB signaling). Hypothalamic inflammation decreases satiety response to insulin and to the adipocyte-derived hormone leptin, which can contribute to positive energy balance and development of obesity [50]. Several cellular mechanisms contribute to hypothalamic aging in healthy and obese individuals, including genomic instability, telomere shortening (replicative senescence), epigenetic mechanisms, stem-cell depletion, endoplasmic reticulum stress, loss of proteostasis, and autophagy [51]. 

In diet-induced obesity, white adipose tissue dysfunction is the primary source of altered levels of circulating free fatty acids, several hormones called adipokines, and proinflammatory cytokines. White adipose tissue depots are in the subcutaneous and visceral compartments. In addition to controlling fuel accumulation, the adipose tissue is an important endocrine organ that releases adipokines, which allow for its effective interaction with several other tissues including CNS, liver, muscle, and pancreas to regulate energy metabolism in an efficient and integrated manner in healthy individuals [52]. 

White adipose tissue depots present a complex cell composition, including the main cell type, adipocytes, but also pre-adipocytes, fibroblasts, mesenchymal cells, immune cells (macrophages, T cells and others), endothelial cells, and smooth muscle cells [52,53]. Adipose tissue cellularity can present alterations depending on whether their metabolic status is lean or obese. In lean adipose tissue, resident or recruited macrophages are mostly M2-anti-inflammatory that produce TGF-β (transforming growth factor beta), IL-10 (interleukin 10), CCL17, 18, 22, and 24 (CC chemokine ligands). In adipose tissue from obese subjects the main macrophage population is the M1-proinflammatory cells that produce mainly IL-6, TNF-α (tumor necrosis factor alpha), IL-1β, and IFN-γ (interferon gamma) [54]. Therefore, these proinflammatory markers produced by adipose tissue may cross BBB to trigger neuroinflammation and brain aging.

Leptin is an important hormone released by adipocytes involved in white adipose tissue and brain crosstalk. Leptin production positively correlates with fat mass. Therefore, obese individuals present with hyperleptinemia, which causes differential modulation of food intake, glucose, and fat metabolism through interaction with hypothalamic receptors [55]. Leptin acts mainly on the arcuate hypothalamic nucleus (Arc), activating anorexigenic neurons that express proopiomelanocortin and cocaine/amphetamine-related transcript (POMC/CART neurons), thus inhibiting orexigenic neurons that express the neurotransmitters neuropeptide Y and agouti-related protein (NPY/AgRP neurons) [56,57]. In lean individuals, leptin action results in decreased food intake and increased energy expenditure to control fat mass expansion by a negative feedback loop. However, in obese individuals, hyperleptinemia is commonly associated with hypothalamic leptin resistance and a progressive increase of adiposity [58]. 

In experimental models of aging, hypothalamic regulation of lifespan has been suggested as it was demonstrated an increased hypothalamic expression of NF-κB pathway in experimental models of advanced age, and that inhibition of this pathway delays aging and extends lifespan of rodents [59,60]. In old rats, brain inflammation induced by LPS has been associated with increased peripheral inflammatory markers and hyperleptinemia, while treatment with anti-leptin serum partially reverses brain inflammation, highlighting the crucial role of leptin as a mediator of brain inflammation in aging. As indicated, dietetic interventions may not only ameliorate metabolic disruption (such as insulin sensitivity), but also regulate the influence of peripheral hormones and mediators in brain “inflammaging”, a process where inflammation is strongly correlated, and possibly causal, to aging phenomena [61].

In humans, the relationship between leptin and cognition in the elderly population is controversial and deserves careful interpretation. While mid-life obesity and systemic metabolic changes, such as high leptin circulating levels, are risk factors in the development of dementia, low plasma leptin levels later in life are associated with worsening cognitive decline and increased risk of developing AD [62,63]. This controversial pattern seems to be age-dependent. Possibly, higher levels of leptin in mid-life could trigger initial deleterious mechanisms in the brain, predisposing it to aging-related diseases, and after the actual development of cognitive impairment in older individuals, changes in the whole body energy metabolism can result in weight loss and, consequently, lower leptin levels. 

In healthy elderly subjects, plasma leptin levels are positively correlated with grey matter volume of several brain regions, including the hippocampus [64], and inversely correlated with aging-related cognitive decline [65]. In a prospective study of the Framingham original cohort, circulating leptin levels were associated with reduced incidence of dementia and AD in asymptomatic older adults [66]. Therefore, these studies suggest a protective effect of leptin on brain function. On the contrary, mild cognitive impairment was positively correlated with serum leptin and IL-1β levels, and inversely correlated with adiponectin in elderly population [67]. Additionally, in elderly individuals included in the Alzheimer’s Disease Neuroimaging Initiative (ADNI) study, higher leptin levels were associated with deficits in frontal, parietal, temporal and occipital lobes, brainstem, and the cerebellum [68]. 

In contrast to obesity and hyperleptinemia, caloric restriction is another energetic challenge that can modulate adiposity, brain function, and lifespan. From the evolutionary perspective, the brain is a unique organ that presents optimal cognitive function performance under hunger/food scarcity conditions [69]. Caloric restriction can optimize brain function throughout several molecular and cellular mechanisms that include modulation of synaptic activity, brain-derived neurotrophic factor (BDNF) signaling, mitochondrial biogenesis, DNA repair, protein homeostasis, and reduced inflammation [70]. Sirtuins are important mediators of the brain metabolic adaptation during caloric restriction. Sirtuins (SIRT1–SIRT7) are enzymes commonly known as NAD+-dependent histone deacetylases (HDAC). However, in addition to controlling gene expression by chromatin remodeling, sirtuins can regulate a variety of cellular functions by modulating the activity of kinases, transcription factors, and other molecular targets [71]. Brain content of SIRT1 increases in response to caloric restriction and is involved in several brain and behavioral adaptations in mice [72,73]. Activation of SIRT1 has been associated with a protective role in neuroinflammation induced by LPS or IL1-β in vivo and in vitro models, as well as in neuroinflammation associated with AD. Interestingly, these studies show that SIRT1 activation may be also induced by dietary components such as resveratrol (a potent antioxidant polyphenol), and eicosapentaenoic acid (EPA) and docosahexaenoic acid (DHA), the major ω-3 polyunsaturated fatty acids (PUFAs) from fish oil [74,75]. 

## 4. Fatty Acids

PUFAs, especially DHA, play an essential role in the maintenance of central and peripheral metabolism. DHA is produced by desaturation and elongation of α-linolenic acid (ALA), which is considered essential in the diet, since mammals are unable to biosynthesize DHA and EPA from precursors with shorter hydrocarbon chains [76]. Humans are required to intake dietary ALA present in leafy vegetables and oil, together with EPA and DHA from fish oil [77]. ALA, DHA, and EPA (i.e., omega-3) should be maintained at appropriate levels in the diet, since the quantitative ratio between linoleic acid (LA, i.e., omega-6) and ALA is critical to control the production of arachidonic acid (ARA) and pro-inflammatory mediators (e.g., eicosanoids), which play an important role in the progression of cardiovascular diseases, diabetes, and brain disorders [78].

Cerebrovascular diseases and neurodegenerative processes are highly dependent on the stability of central blood. The proper functioning of reperfusion systems attenuates cell death and prevents stroke episodes, resulting in less cognitive impairment over time [79,80]. Maintenance of the connective brain structure in patients with AD is one of the major challenges in preserving memory and associated functions; changes such as severe hippocampal atrophy and increased lesions in white matter are, at least, prevented by interventions in which PUFAs-enriched diets are provided, especially DHA and EPA. In addition, patients undergoing diets rich in these fatty acids are less likely to develop neurodegenerative processes or functional and cognitive loss toward the progression of the disease [81].

In a recent study, senescent rodents depleted of omega-3 had greater dysfunction in glutamatergic synapses and 30% lower uptake of glutamate in astroglia from CA1 hippocampal area [82]. Studies using imaging methods have shown that, even in individuals with normal cognition, fish oil supplementation is positively associated with a greater average volume of the hippocampus, cingulate cortex, and orbitofrontal areas. Fish oil supplementation was also related to higher scores on standardized cognitive tests. Presence of the ApoE4 allele seems to be a determining factor in the outcome of clinical trials with DHA, since patients without this allele present better results from dietary and pharmacological interventions using omega-3 [83,84]. 

Omega-3 fatty acids decrease the synthesis of proinflammatory lipid mediators produced by the omega-6 and ARA metabolism in a competitive manner. Omega-3 acts as endogenous ligand of the transcriptional factors peroxisome proliferator-activated receptors (PPAR-γ and α) that attenuate the activity of NF-κB mediated inflammatory pathways (e.g., cycloxygenase-2, TNF, IL-1) and modulate the mechanism of fatty acid oxidation, peroxisome proliferation, sensitization to insulin, and adipocyte differentiation, a potential therapeutic target in the treatment of dyslipidemia [85,86]. In the brain, PPAR-γ participates in many aspects of microglial activation, myelination, heat shock protein (HSP) response, cell death, production of TNF-α, and inhibition of Activator Protein 1 (AP-1) and NF-κB, besides reducing the synthesis of nitric oxide (NO) and prostaglandin E2 (PGE2). Therefore, PPAR-γ plays a critical anti-inflammatory role in diseases such as Parkinson’s disease, multiple sclerosis, and AD [87,88]. PPAR-γ has also been demonstrated to be effective in preventing intracerebral ischemic damage, especially in patients with associated morbidities, such as type II diabetes [89]. 

Afshordel and colleagues (2015) have recently explored another central mechanism of DHA, which can be converted to neuroprotectin D-1 (NPD-1), an unesterified derivative with neuroprotective properties. Authors showed that fish oil supplementation in aged rodents can raise levels of unesterified DHA and NPD-1-like metabolites in parallel to increased Bcl-2 levels in the brain, suggesting that EPA and/or DHA contribute to the control of apoptotic mechanisms and mitochondrial function [90].

Omega 3 fatty acids play an important role in preventing chronic injuries in peripheral and central metabolism, especially for patients undergoing Western diets. In fact, recent data on in vivo models suggest that supplementation of these fatty acids can prevent cognitive decline, promote hippocampal protection, and neuroplasticity [91]. The balance between saturated and unsaturated fatty acids may control features of the peripheral metabolism. Kaplan and Greenwood discuss the importance of SFA consumption on the control of feeding behavior in animal models, highlighting its negative influence on the hepatic metabolism of glucose, which in turn regulates its availability to the brain, where it can control the production of neurotransmitters, trophic factors, feeding behavior, and general cognitive performance [92].

The benefits of consuming low-calorie meals, fibers, and omega-3 rich foods are well supported by the literature [60,77,78,79,80,81,82]. Eating patterns, however, depend on the individual’s ability to control dietary intake. For subjects undergoing nutritional counseling, adherence to prescribed nutritional programs greatly varies (13–76%) according to how complex and deep is the involvement of the patient with inadequate eating habits [93]. A recent study investigated the role of SFA on feeding behavior, and epidemiological and experimental data suggest that the indiscriminate consumption of SFA and simple sugars promotes damage in hippocampal regions involved in negative control of appetite and cognitive processing of reward [94].

Finally, glucolipotoxicity describes the synergistic effect of glucose and SFA on the induction of apoptosis in human β pancreatic cells, and the presence of an omega-6 polyunsaturated (LA) or monounsaturated (i.e., oleic acid) fatty acid reduces this toxicity [95]. Several authors have demonstrated the deleterious effect of glucolipotoxicity on pancreatic β cells, highlighting its role in the progression of type II diabetes, mitochondrial dysfunction, production of ROS, and deposition of cholesterol and ceramide in β cells [96,97,98]. Novel therapeutic targets for the treatment of type II diabetes now consider the strong synergistic effect of SFA and glucose in progression of the disease [99,100].

Together, these data demonstrate the important neuroprotective role of PUFAs, attenuating the deleterious effects of excessive omega-6 and SFA consumption in obesity induced by Western diets, and demonstrating the negative impacts of glucolipotoxicity. Intake of the correct amount of fatty acids and carbohydrates plays an essential role in the aging process, neuroinflammation, AD, and other neurodegenerative diseases [101].

## 5. Antioxidants

Central degenerative processes are importantly linked to the excessive production of ROS, which promotes oxidative damage to proteins, lipids, and nucleotides, causing connective and vascular disorders, loss of neuronal content, activation of microglia/macrophages, and induction of mechanisms preceding the onset of AD. The use of antioxidants, such as ascorbic acid (AAC) and vitamin E (VE), has been shown to be effective in combating the symptoms of cognitive loss and oxidative stress [102]. 

Humans and primates have lost the ability to synthesize AAC due to absence of the gene coding for L-gulono-γ-lactone oxidase enzyme (i.e., Gulo), which converts L-gulonolactone into L-ascorbic acid. In animals expressing this enzyme, inactivation of the Gulo gene implies the need for antioxidant supplementation even prenatally, becoming required for survival. If supplementation of AAC is removed, the subjects become anemic, lose weight, and die, presenting damage to vascular integrity, proliferation of smooth muscle cells, and increased oxidative stress, which recruits compensatory antioxidant mechanisms [103,104]. In humans, consumption of approximately 10 mg/day of AAC is enough to prevent the onset of deficiency symptoms [105]. 

AAC transport into the brain is mediated by the sodium-vitamin C co-transporters 2 (SVCT2), ensuring a sharp concentration gradient through the choroid plexus [106]. Although not responsible for the central concentrations of AAC, SVCT1 transporters are essential for the maintenance of plasma levels of the antioxidant, which in turn modulates the availability of AAC into the cerebrospinal fluid (CSF) and ultimately to the brain. 

After cerebrovascular disorders, such as transient ischemia and stroke, AAC absorption and SVCT2 expression rises significantly, especially in capillary endothelial cells located in the ischemic region, indicating that AAC is involved in neutralization of ROS produced by the oxidative stress or specifically due to macrophage activity in the damaged region [107]. Lin et al. (2010) showed that intraperitoneal injections of AAC (500 mg/kg in phosphate buffered saline), following compression of the somatosensory cortex of rats, prevented disruption of the BBB and maintained the integrity of the sensory system [108]. This preservation phenomenon may be extended to other types of BBB damage or cerebrovascular disorders that occur during aging process [109].

Recently, it has been proposed that AAC is involved in prevention of cognitive decay and depression in in vivo models, primarily in situations where damage is promoted by oxidative stress or pro-oxidant agents [110,111]. In a cohort study with 117 elderly individuals, the supplementation of AAC was associated with a lower incidence of severe cognitive impairment, with no effect on verbal ability [112]. Guidi and colleagues (2006) evaluated plasma levels of homocysteine (tHcy), a marker of ROS and total antioxidant capacity, in AD elderly patients with either mild cognitive impairment or vascular dementia. Data obtained showed high levels of tHcy and reduced total antioxidant capacity in AD and mild cognitive impairment patients. tHcy levels were also high in vascular dementia patients, while lower total antioxidant capacity was exclusively related to AD individuals. ROS levels were homogenous between groups, indicating that the loss of total antioxidant capacity may be related to progression of cognitive complications [113].

Besides the isolated supplementation of AAC, population studies seek to highlight the participation of other dietary components in preventing cognitive/motor impairment and AD progression. In a study from Morris and colleagues, consumption of antioxidant nutrients, VE, AAC, and β-carotene was investigated in relation to the incidence of AD in a population of individuals aged over 65 years. In this study, only dietary intake of VE was associated with reduced risk of AD; surprisingly, this relationship was observed only in subjects without the allele ApoE4 [114]. Determining the contribution of a specific antioxidant is, however, a difficult task, as these and other phytochemicals apparently act synergistically when present in foods and complex phytoextracts [115].

Another antioxidant intrinsically involved in the metabolic signs of aging and in pathological dynamics of neurodegenerative diseases is glutathione (GSH), a tripeptide composed of glutamic acid, cysteine, and glycine residues. GSH is the most prevalent thiol compound in cells from virtually all body tissues. GSH is essential for cell proliferation, participates in apoptotic processes and ROS neutralization, and also maintains the reduced form of intracellular protein’s sulfhydryl groups [116]. In the brain, GSH is found in higher concentrations in the glial cells, while in neurons the concentrations are slightly lower [117].

GSH is involved in the prevention of mitochondrial damage, cell death, and in the pathogenesis of CNS, providing evidence for the relationship between GSH and diseases such as PD and AD [118,119]. Elucidating the complexity of the neuroprotective mechanisms performed by GSH, in a recent study, it was shown that even non-toxic decreases in GSH concentrations are able to cause an imbalance in NO activity, allowing nitration of proteins, a predictive marker for neurodegenerative diseases [120].

**Figure 1:**
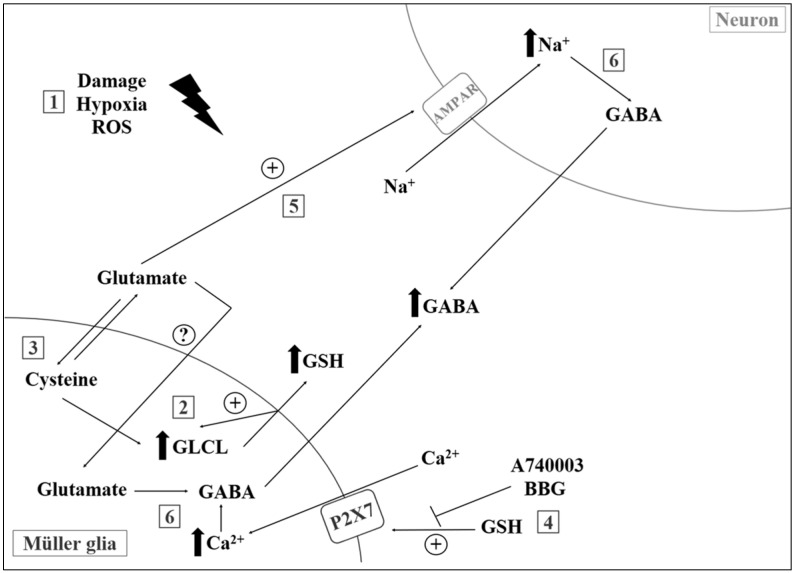
Mechanisms of functional compartmentalization mediated by glutathione in the retinal environment. Tissue damage, hypoxia, and reactive oxygen species (ROS) (1) promote increased activity of antioxidant system intermediates in Müller glial cells, such as γ-glutamylcysteine ligase (GLCL), which stimulates the synthesis/release of glutathione (GSH) (2) and the uptake of cysteine through a glutamate-cysteine antiporter system (3) When released, GSH is capable of activating P2X7 receptors, allowing for intense Ca^2+^ increase in the Müller cells (4) while extracellular glutamate promotes activation of AMPA receptors (α-amino-3-hydroxy-5-methyl-4-isoxazolepropionic acid receptors, or AMPAR) in retinal neurons, leading to higher Na^+^ levels in these cells (5) Finally, intracellular Ca^2+^ (glia) and Na^+^ (neurons) stimulate GABA (gamma-aminobutyric acid) release to the extracellular environment (6). (+) Activation/stimulus. (?) Unknown mechanism.

Attenuation of central levels of GSH, especially in the mitochondria, appears to be a strong indicator of oxidative damage during aging [121]. In a recent work with proton magnetic resonance spectroscopy, authors showed depletion of GSH, increase in lactate, and unchanged levels of AAC in the occipital cortex of elderly compared to young individuals [122]. In another study, Mandal and colleagues showed a linear reduction of GSH concentrations in the frontal cortex during aging, mild cognitive impairment, and diagnosed AD, with gender-specific components [123]. Lower GSH levels were also observed (post-mortem samples) in patients with autism, bipolar disorder, major depression, and schizophrenia [124,125]. Finally, recent investigations from our group suggest that GSH may also act as a signaling molecule in CNS (Figure 1), regulating purinergic activity, ion channel opening, and GABA release. Incubation with milimolar concentrations of GSH induces an acute increase in intracellular calcium levels ([Ca^2+^]_i_) and may act in accordance with reducing properties of GSH during disease and tissue injury [126,127].

## 6. Physical Activity

Regular physical activity has several beneficial effects on health, and exercise capacity is a strong and independent predictor of morbidity and mortality for patients of all ages [128,129]. Over the last decades, life expectancy has been increasing, and the continuous reduction in mortality rates among the elderly population is associated with dietary factors and exercise [130]. In fact, exercise not only can improve life expectancy but can also slow down, delay, or prevent many age-associated chronic pathologies, extending healthspan for an optimal longevity [131,132]. Physical activity can also reverse or attenuate the progression of brain aging, being associated with positive vascular, structural, and neuromolecular changes, including insulin resistance, inflammation, and oxidative stress, which contribute to cognitive decline and brain-related diseases [133,134].

### 6.1. Physical Activity and Disorders of Cerebral Blood Flow

The cerebral blood flow is tightly coupled to the cerebral metabolic rate and neuronal metabolism; thus, systemic vascular dysfunction associated with brain hypoperfusion can compromise cognitive performance [135,136]. Injuries in endothelium and central/peripheral vascular structure involve increased inflammation and oxidative stress [133]. In addition, cerebral blood flow declines with age [137,138], which strongly contributes to the decrease in cognitive function in the elderly [139]. Exercise, in contrast, increases cerebral blood flow in an intensity-dependent manner and has been shown to improve cognitive function and brain aging [137,140]. Aged mice presented lower cerebral blood flow, accompanied by a lower content of endothelial nitric oxide synthase (e-NOS) and vascular endothelial growth factor (VEGF) in the brain microvasculature, when compared to young mice; training in aged mice improved these functional parameters [141]. Mice submitted to running exercise exhibit reduced cerebral lesion sizes after a cerebral ischemia episode, and this effect was blunted in the e-NOS deficient mice. Running also improved functional outcome associated with higher cerebral blood flow and angiogenesis in the ischemic striatum, which was completely abrogated in animals treated with L-NAME (*N* omega-nitro-L-arginine methyl ester), a NOS inhibitor. These data indicate that exercise improves long-term stroke outcome via NO-dependent mechanisms [142].

In an animal model of vascular dementia induced by bilateral carotid artery occlusion, treadmill exercise reduced the memory impairment caused by the chronic cerebral hypoperfusion and induced hippocampal neurogenesis via the BDNF-pCREB pathway [143]. Imaging analyses conducted both in mice and in young/middle-aged humans showed that exercise-induced neurogenesis associated with increased cerebral blood volume occurs selectively at the hippocampal dentate gyrus [144,145]. Similarly, a study conducted in healthy older humans (60–77 years) also observed that aerobic fitness improvement was associated with positive changes in hippocampal perfusion and early recall and recognition memory; however, these benefits decrease with progressing age, indicating that the capacity for vascular hippocampal plasticity may be age-dependent [146].

### 6.2. Brain Volume and the Effect of Physical Activity on Brain Atrophy

Aging-related brain atrophy is commonly associated with cognitive impairment and memory loss. In fact, the rate, extent, and brain regions showing atrophy can vary among individuals [145]. A recent study by Hanning and colleagues [147] found that brain atrophy in the elderly is associated with higher IL-6 and IL-8 circulating levels, suggesting a role for systemic inflammation in the brain atrophy pathogenesis. Greater brain volumes are associated with greater cognitive reserve and a higher capacity to deal with AD pathology without the clinical manifestation of cognitive impairment [140]. In individuals at the age of 75 years, a higher level of physical activity was associated with better memory performance and with greater volumes of both total brain and white matter [148,149]. In addition, higher aerobic fitness level was related to higher hippocampal volume and better memory performance in older non-demented individuals [149], older individuals in the earliest stages of AD [150], and in preadolescent children [151], highlighting the impact of physical activity in increasing brain volume of individuals from all ages. Interestingly, a 42-year follow-up study identified that men with high cardiovascular fitness at age 18 had a lower risk of early-onset dementia and mild cognitive impairment later in life [152].

### 6.3. Sexual Dimorphism and Physical Activity

Sexual dimorphism is observed in brain anatomical structures, neurochemicals, and functions, and not surprisingly men and women also differ in the incidence and nature of CNS-related diseases, such as cognitive impairment, AD, autism, schizophrenia, and eating disorders [153]. In addition, females exhibit stronger immune response, improved antioxidant capacity, better redox, and functional state of their immune cells and, accordingly, the “inflammaging” process in the elderly shows gender differences, including higher serum levels of IL-6 in men than in women [154,155]. Elderly individuals with mild cognitive impairment have higher mortality rates, compared with cognitively normal age-matched individuals, and the mortality rate was highest in men [156]. Although cerebral blood flow decreases with age, women have higher levels than men in all ages [157]. The human male brain exhibits more global gene expression changes than the female brain throughout aging, with gene expression mostly down-regulated to 60 years old in men. On the other hand, among older ages, women showed progressively more gene expression changes than men. Interestingly, the major category of down-regulated genes in men was related to protein processing and energy generation [158].

Not surprisingly, exercise impact between genders is also different, and is explored in mixed gender studies. Overall, studies comparing male and female indicate that the positive effect of physical activity or exercise on brain volume, cognition, and AD risk is more pronounced in females [159,160]. However, this subject remains controversial. It was observed that cardiorespiratory fitness was positively associated with total and cortical gray matter volumes in elderly men at increased risk for AD [161]. This profile was not observed in women, and authors suggested that cardiorespiratory fitness might be beneficial to brain health, only in men, at the age of 60 years and older. 

### 6.4. Physical Activity and Brain Disorders of Metabolic Origin

Insulin is also an important player in the control of degenerative scenarios. In addition to the modulation of energy metabolism, it regulates several features that are essential for healthy aging: cerebral blood flow, inflammatory responses, oxidative stress, Aβ (amyloid beta oligomers) clearance, tau phosphorylation, apoptosis, synaptic plasticity, and memory formation [162]. In humans, insulin resistance and type 2 diabetes have been shown to predict the development of aging-related diseases and a preserved insulin action is strongly associated with longevity [163,164]. AD development and symptoms are closely related to an insulin-resistant brain state, and type 2 diabetes mellitus is a risk factor for dementia and AD [165]. Intranasal insulin therapy in patients with AD or mild cognitive impairment has been associated with improvement in cognitive function [166,167,168], increased brain volume, including hippocampus, and reduction in the tau-P181/Aβ42 ratio [167].

Exercise can stimulate cellular insulin signaling and sensitivity in peripheral organs [169] and in the brain, with a beneficial impact on brain structure [170] and function [171,172]. A major factor for the development of insulin resistance is obesity [173], and the impact of obesity on unhealthy brain aging has been discussed previously in this review. Exercise is an effective intervention to prevent or treat obesity and obesity-related insulin resistance [174] and improve adipokine profile in obese individuals through the increase of adiponectin and the reduction of hyperleptinemia [175,176,177]. In addition, the exercise-induced hippocampal neurogenesis was remarkably attenuated in adiponectin-deficient mice, highlighting that adiponectin may be an essential factor mediating this effect via its receptor 1 (adiponectin receptor 1, or ADNR1) and AMPK (5′ AMP-activated protein kinase) activation [178]. 

### 6.5. Inflammation and a Role for Physical Activity

Exercise induces insulin sensitivity and glucose disposal through several pathways, including improvements in inflammation and oxidative stress that are high-risk factors for cognitive impairment and accelerated aging [163,165]. In elderly individuals of both sexes, exercise improves inflammatory profile by reducing serum markers, such as C-reactive protein, IL-6, and TNF-α [177,179,180,181,182]. In peripheral blood mononuclear cells obtained from aged individuals, exercise training induced lower protein expression of toll-like receptors (TLR2 and TLR4) associated with an anti-inflammatory status linked to myeloid differentiation primary response gene 88 (MyD88)-dependent and MyD88-independent pathways [182]. Additionally, the exercise-induced improvement in inflammatory profile in the elderly was associated with positive changes in cognition [183] and greater total brain volume [184]. In young healthy mice, exercise did not promote changes in serum inflammatory profile; however, reductions in IL-6 and TNF-α were induced in the hippocampus, indicating that it can promote an anti-inflammatory effect in the brain without affecting the peripheral cytokines production [185]. Although exercise promotes several long-term benefits, including in the proinflammatory state, the acute exercise responses are associated with increased serum levels and tissue expression of IL-6 and TNF-α [186,187]. In a mouse model of traumatic brain injury associated with neurodegeneration and chronic neuroinflammation, it was observed that delayed exercise onset (5 weeks after trauma) caused improvements in working and retention memory, decreased lesion volume, increased neurogenesis in the hippocampus, and reduced IL-1b gene expression. However, these improvements were not observed when exercise was initiated 1 week after the brain injury. In fact, it exacerbated chronic classical inflammatory responses, highlighting the importance of timing of exercise onset and its relation to cognitive outcomes and neuroinflammation [188].

### 6.6. A Role for Physical Activity in Autophagy and Oxi-Inflammaging

Autophagy is a physiological and catabolic process, vital for the maintenance of cell homeostasis by effectively clearing dysfunctional organelles such as damaged mitochondria and malformed proteins. Disrupted autophagy, however, contributes to unhealthy aging and decreased longevity [189,190]. Elderly individuals submitted to exercise training exhibit increased expression of autophagy related-genes, including beclin-1, Atg12, Atg16, and the LC3II/I in peripheral blood mononuclear cells compared with sedentary individuals [191,192]. In addition, the expression of NLRP3, Bcl-2, and Bcl-xL was reduced in peripheral blood mononuclear cells of trained elderly individuals, indicating improvement in autophagy, prevention of NLRP3 inflammasome activation, and reduction of apoptosis [191].

Several studies have revealed that physical activity or exercise elicits a combined effect improving the redox state and enhancing inflammatory defenses, combating the “oxi-inflamm-aging” process [132,155]. Healthy-aged female rats submitted to long-term exercise training showed lower ROS content, lower protein carbonyl content, and increased SOD 1 and SOD 2 (superoxide dismutase 1 and 2) protein expression in the hippocampus compared with sedentary age-matched rats, indicating a beneficial effect on the oxidative status [193]. In an aged mice model of AD (3xTg-AD), voluntary exercise reversed lipoperoxidation and oxidized glutathione levels, while improving the antioxidant enzyme CuZn-SOD content in the cerebral cortex. These changes were associated with optimized behavior and cognition, and reduced amyloid/tau pathology, highlighting the neuroprotective effect of exercise through regulation of redox homeostasis [194]. Neuronal mitochondria are especially susceptible to oxidative stress; therefore, the beneficial impact of exercise on redox balance has many positive effects on mitochondrial function [195]. In young and aged rats, exercise induced a reduction in oxidative stress accompanied by increased mitochondrial biogenesis, dynamics and mitophagy in the brain [193,196].

### 6.7. Antineurodegenerative Effect of Muscle Metabolites

Physical activity and exercise directly affect skeletal muscle physiology. Skeletal muscle is a metabolically active tissue that releases myokines, which might be involved in the beneficial effects of exercise [197,198]. Important neural factors associated with neurogenesis, angiogenesis, and cognition, such as BDNF and VEGF, are also produced by skeletal muscle and modulated by exercise. Indeed, the significance of these factors released by the skeletal muscle during exercise to the brain physiology is still unclear. In both young and elderly individuals, the skeletal muscle BDNF expression and the serum concentration of BDNF increase after exercise, and they were associated with structural and functional benefits to the brain [198,199,200]. However, it has been proposed that the brain contributes with 70–80% of circulating BDNF both at rest and during exercise; therefore, the systemic impact of the BDNF released from the muscle needs further investigation [201]. 

Finally, irisin is an exercise-induced myokine that is highly expressed in the brain [197,202]. Interestingly, the knockdown of the precursor of irisin, FNDC5, in neuronal precursors impaired their development into mature neurons [203]. Since the FNDC5 expression in the brain is upregulated with exercise, the specific tissue contribution to the beneficial effect of exercise on the brain is still to be defined [202].

## 7. Conclusions

**Figure 2:**
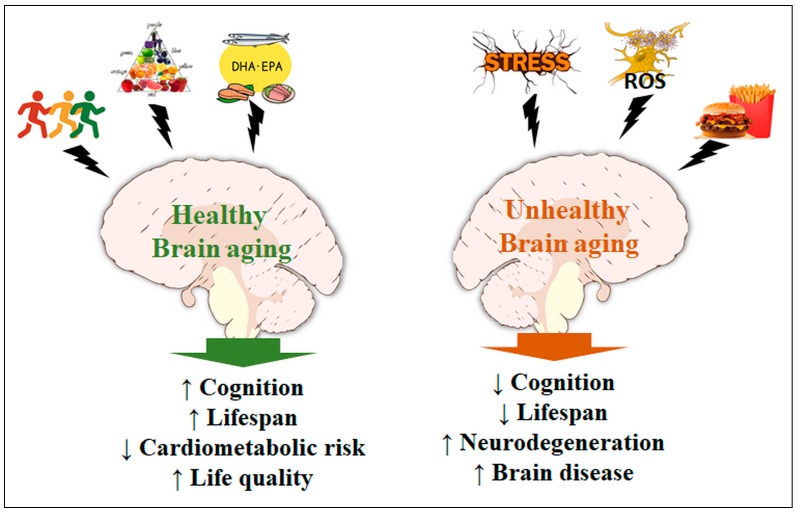
Brain dynamics in healthy and unhealthy aging. Moderate physical activity, low-calorie diets, and essential fatty acids are amongst the main elements of a healthy brain, where we observe less or no cognitive decline, greater lifespan, reduced cardiovascular (and metabolic) risks, and thus overall better quality of life. Conversely, a continuously stressed brain, either by an unstable environment or by chemical mediators (e.g., ROS, reactive nitrogen species and other radicals) will suffer from cognitive decline and increased risk for neurodegeneration and other brain diseases, while affecting the individual’s lifespan. Also, high caloric meals and/or typical cafeteria diets are risk factors for the development of several such affections. (↑) Increased. (↓) Decreased.

Aging is a sensitive process for the maintenance of the metabolic and functional balance of the brain. When compiled, data indicate the complexity of action and the necessity of various dietary/endogenous antioxidants, in addition to the proper balance in the consumption of essential fatty acids (omega-3 and -6) and physical activity, whose synergistic actions allow for the maintenance of physiological conditions, even during severe metabolic stress (Figure 2). Recent investigations aim to elucidate mechanisms for preventing the intrinsic effects of the aging process in afflictions such as ischemic disorders [204] and the functional decay of mitochondria [205]. However, finding pharmacological or dietary resources capable of significantly intervening with the neurodegenerative processes remains a great challenge, and several groups are currently in a collective effort to narrow the therapeutic spectrum into a more realistic set of approaches [206,207,208]. Future research should rely on novel integrative methods present in systems biology, which allows for a broad analysis of the metabolic interactions in aging and disease processes.
